# Construction of a linezolid-resistant strain of methicillin-susceptible *Staphylococcus aureus* and its multi-omics based mechanism study

**DOI:** 10.3389/fcimb.2026.1839912

**Published:** 2026-06-29

**Authors:** Wei Zhang, Linhui Huang, Yanye Tu, Feng Wang, Hong Li, Yanzi Chang, Xudong Feng, Jie Zhou

**Affiliations:** 1 Department of Clinical Laboratory, The Affiliated Li Huili Hospital of Ningbo University, Ningbo, Zhejiang, China; 2 School of Medicine, Ningbo University, Ningbo, Zhejiang, China

**Keywords:** linezolid, metabolomics, methicillin-susceptible Staphylococcus aureus, molecular mechanism, resistance induction, transcriptomics

## Abstract

**Background:**

The emergence of linezolid−resistant methicillin−susceptible Staphylococcus aureus (MSSA) is a growing clinical threat, but the underlying mechanisms are unclear. Here, we generated stable resistant derivatives and used a multi−omics strategy to investigate the transcriptional and metabolic regulation underlying linezolid resistance in MSSA.

**Methods:**

A clinical isolate of linezolid-susceptible *S. aureus* (strain *S. aureus*_97) was used as the parental strain. A stable linezolid-resistant derivative (designated LZR) was generated through stepwise induction with subinhibitory concentrations of linezolid. The genetic stability of the resistant phenotype was subsequently confirmed via phenotypic stability assays. To elucidate the mechanisms underlying resistance, comparative transcriptomic (RNA-seq) and untargeted metabolomic (LC-MS/MS) analyses were performed to identify differential gene expression and metabolic profiles between the resistant (LZR) and susceptible (LSS) strains.

**Results:**

The successfully induced linezolid-resistant derivative (LZR) exhibited a 16-fold increase in minimum inhibitory concentration (MIC) against linezolid (32 μg/mL) compared with the parental strain (2 μg/mL), and this resistant phenotype remained stable after 50 serial passages in antibiotic-free medium. CCCP was observed to reduce the MIC of LZR in a concentration-dependent manner. Transcriptomic and metabolomic analyses revealed widespread transcriptional reprogramming and metabolic perturbations in the resistant strain. Differentially expressed genes were mainly enriched in pathways associated with oxidative phosphorylation, ribosome function, and glycerolipid metabolism, while differential metabolites were primarily involved in the biosynthesis of secondary metabolites and amino acid metabolism. Integrated analysis identified key co-enriched pathways with distinct regulatory patterns, which collectively remodeled bacterial material and energy metabolism and were closely associated with the emergence of linezolid resistance.

**Conclusion:**

A stable linezolid-resistant MSSA strain was developed in this work, revealing an intricate network of regulatory interactions that drive resistance at both the transcriptional and metabolic levels. These findings provide an experimental foundation and theoretical basis for further understanding the mechanisms of linezolid resistance in MSSA and for developing strategies to combat resistance.

## 1 Introduction

*Staphylococcus aureus* (*S. aureus*) is an opportunistic pathogen that colonizes the skin, mucous membranes, and environmental surfaces in humans. It can cause a range of infections, from skin and soft tissue infections to community-acquired pneumonia and can progress to life-threatening conditions such as sepsis and infective endocarditis in severe cases. Consequently, it remains a major public health concern worldwide (Liu et al., 2024a). While methicillin-sensitive *Staphylococcus aureus* (MSSA) remains susceptible to beta-lactam antibiotics such as methicillin, its resistance to other classes of antimicrobial agents has been progressively increasing due to the widespread use of these drugs in clinical practice. This trend poses significant challenges to the clinical management of infections caused by this pathogen (Sanchini, 2022).

Linezolid, the first synthetic oxazolidinone antibiotic, has become a cornerstone in the treatment of Gram-positive bacterial infections due to its unique mechanism of action, particularly in managing infections caused by multidrug-resistant strains. It exerts its antibacterial effect by specifically binding to the 23S rRNA of the bacterial 50S ribosomal subunit, thereby preventing the formation of a functional 70S initiation complex and potently inhibiting bacterial protein synthesis. This distinctive mechanism also confers a low potential for cross-resistance to other antimicrobial classes, making it a widely used agent in the clinical management of moderate-to-severe infections caused by multidrug-resistant Gram-positive pathogens (Hashemian et al., 2018). However, with the increasingly widespread clinical use of linezolid, particularly in the long-term treatment of critically ill patients and immunocompromised populations, linezolid-resistant *Staphylococcus aureus* strains have continued to emerge. This trend has markedly increased clinical treatment failure rates and the risk of patient mortality, thereby severely compromising the clinical utility of linezolid (Yang et al., 2025; Folan et al., 2018).

It is well established that the mechanisms of linezolid resistance in *Staphylococcus aureus* primarily fall into three categories: ribosomal target site modification, altered efflux pump expression, and regulatory adaptations in metabolism. Ribosomal target-site modification is the predominant mechanism. Linezolid reportedly exerts its antibacterial effect by binding to the peptidyl transferase center of the bacterial 50S ribosomal subunit. Mutations such as G2576T in domain V of the 23S rRNA gene can directly alter the spatial configuration of this target site, thereby reducing the drug’s binding affinity. Besides, mutations in the *rplC* and *rplD* genes, which encode ribosomal proteins uL3 and uL4, respectively, can indirectly compromise the structural stability of the 23S rRNA, further diminishing linezolid binding affinity (Yang et al., 2025). The *cfr* gene-mediated methylation of 23S rRNA confers resistance not only to linezolid but also to several other antimicrobial classes, thereby leading to a multidrug resistance phenotype (Aleksandrova et al., 2023). Altered efflux pump expression represents another key mechanism of resistance. Overexpression of efflux pump genes such as *norA*, *norB*, and *lmrS* can reduce intracellular linezolid concentrations by enhancing drug efflux, thereby preventing the antibiotic from reaching effective inhibitory levels (Hassanzadeh et al., 2017; Ojha et al., 2025). Overexpression of the *MmpS-MmpL* efflux pump has been closely associated with linezolid resistance (Zhang et al., 2025). In addition, the regulatory role of bacterial metabolic reprogramming in the development of resistance has emerged as a research focus in recent years. Bacteria can enhance their capacity to establish a resistant phenotype by modulating key metabolic processes such as energy metabolism and amino acid biosynthesis (Zhang et al., 2024b). Concurrently, metabolic reprogramming may also reduce basal respiration rates, thereby preventing antibiotic-induced dysregulation of tricarboxylic acid cycle activity and minimizing the accumulation of metabolic toxicity, ultimately attenuating the drug’s lethal effects (Lopatkin et al., 2021). It has been previously demonstrated that in *Staphylococcus aureus* exposed to linezolid, core metabolic pathways, including amino acid biosynthesis and the tricarboxylic acid (TCA) cycle, undergo significant fluctuations, and nucleotide metabolism, particularly the pyrimidine synthesis pathway, is also markedly affected (Luo et al., 2025a). These metabolic alterations are primarily characterized by the downregulation of energy metabolism and the upregulation of amino acid metabolism, and the interplay between these processes contributes to the modulation of linezolid resistance in this pathogen. However, the specific mechanisms underlying MSSA metabolic adaptation in response to linezolid induction, as well as the coordinated regulatory networks operating at the transcriptional and metabolic levels, have not yet been systematically elucidated. Furthermore, molecular biology studies conducted in a single dimension cannot fully reveal the complex regulatory mechanisms underlying the development of resistance, underscoring the urgent need for integrated multidimensional investigations.

Transcriptomics enables the systematic analysis of global changes in gene expression in bacteria under drug pressure, facilitating the identification of differentially expressed genes and core regulatory pathways associated with resistance. Metabolomics, on the other hand, enables precise characterization of dynamic metabolite profiles, thereby directly reflecting bacterial metabolic adaptation strategies. The integrated analysis of these two approaches can establish a comprehensive “gene-metabolite” regulatory network, enabling elucidation of resistance mechanisms from both molecular regulatory and metabolic phenotypic perspectives, thereby offering a more comprehensive view for such investigations. Currently, multi-omics studies on linezolid-resistant *Staphylococcus aureus* have predominantly focused on methicillin-resistant *S. aureus* (MRSA), with relatively limited investigation into MSSA. However, as one of the most isolated strains in clinical infections, MSSA exhibits both specific characteristics and general principles underlying the mechanisms of linezolid resistance that warrant further exploration.

Based on the aforementioned findings, the present study aimed to investigate the molecular mechanisms and metabolic adaptation strategies underlying linezolid-induced resistance in MSSA. A clinical isolate of linezolid-susceptible MSSA (strain *S. aureus*_97) was used as the parental strain, and a stable linezolid-resistant derivative was generated through stepwise induction with subinhibitory concentrations of the antibiotic. Integrative transcriptomic and untargeted metabolomic analyses were subsequently performed to systematically identify differentially expressed genes, differential metabolites, and key regulatory pathways between the resistant and susceptible strains. Our findings are expected to provide a theoretical basis for the early detection of and intervention in clinical linezolid resistance, as well as to inform the development of novel anti-*S. aureus* agents by identifying potential molecular targets.

## 2 Materials and methods

### 2.1 Source and the identification of strain

The experimental strain used in this study was a linezolid-susceptible (LSS) *Staphylococcus aureus* strain (designated *S. aureus*_97), isolated from a clinical sample obtained at the Li Huili Hospital, affiliated with Ningbo University. The strain was assigned the laboratory identification number 97 and preserved at -80 °C in an ultra-low-temperature freezer for subsequent use. Species identification and purity verification were performed using the EXS3600 automated microbial mass spectrometry system (Zybio, China) in conjunction with the VITEK 2 Compact automated microbial analysis system (bioMérieux, France) to ensure the absence of contamination and accurate identification. The study protocol was approved by the Medical Ethics Committee of Li Huili Hospital, Ningbo Medical Center(Approval No. KY2025SL010-01), and all experimental procedures were conducted in accordance with clinical ethical standards.

### 2.2 Antimicrobial susceptibility testing

Antimicrobial susceptibility testing was performed using the VITEK 2 Compact automated system, with broth microdilution, to determine the minimum inhibitory concentration (MIC) of linezolid against the S. aureus isolate. The broth microdilution assay was conducted strictly according to the manufacturer’s instructions provided with the linezolid susceptibility testing kit(Wenzhou Kangtai Biotechnology Co., Ltd., China; Lot No. YF2018J3). In brief, the test strain was cultured to the logarithmic growth phase, and fresh single colonies were used to prepare a bacterial suspension adjusted to a 0.5 McFarland standard. A 10 µL aliquot of this suspension was then added to 2 mL of commercial culture broth (1:200), and the mixture was thoroughly mixed. Subsequently, 100 µL of this diluted bacterial suspension was inoculated into each well of a microtiter plate containing 12 two-fold serial dilutions of linezolid (concentration range: 0.064-128 µg/mL). The microtiter plate was incubated aerobically at 35-37 °C for 16–20 hours. MIC values were interpreted strictly in accordance with the kit instructions to ensure the reliability of the results.

### 2.3 *In vitro* induction of resistance

A clinical MSSA strain with an initial linezolid MIC of 2 µg/mL was selected as the parental strain for *in vitro* resistance induction and designated *S. aureus*_97 (Linezolid-Susceptible, LSS). The strain was first streaked onto Columbia blood agar plates and incubated at 37 °C for 18–24 hours to obtain pure cultures. Fresh single colonies were then collected from the agar plates to prepare a bacterial suspension adjusted to a 0.5 McFarland standard. A 30 µL aliquot of this suspension was inoculated into 3 mL of Luria-Bertani (LB) broth and incubated at 37 °C with constant shaking for 18–24 hours to achieve logarithmic growth phase, ensuring consistent metabolic activity of the bacteria during subsequent induction procedures. For resistance induction, 30 µL of the *S. aureus*_97 suspension in the logarithmic growth phase was transferred into LB broth containing a subinhibitory concentration (1/4× MIC) of linezolid (Shanghai Aladdin Biochemical Technology Co., Ltd., China; Lot No. J2423220), and the mixture was incubated at 37 °C with shaking. Serial passaging was performed 4–8 times at each drug concentration to accumulate resistance-associated mutations. To exclude potential cross-contamination during serial passaging, cultures from each passage were streaked onto Columbia blood agar plates, and single colonies were identified using the EXS3600 automated microbial mass spectrometry system (Zybio, China) to confirm strain consistency. After every 4 passages, linezolid susceptibility testing was performed using the broth microdilution method described above. Once bacterial growth was confirmed at the current concentration, the strain was progressively transferred into LB broth containing increasing concentrations of linezolid (1/2× MIC, 1× MIC, 2× MIC, 4× MIC, 8× MIC, 16× MIC, and 32× MIC). This process of serial passaging and stepwise escalation of concentration was repeated until the induced strain consistently exhibited a linezolid MIC ≥8 µg/mL (Aggarwal et al., 2024).

### 2.4 Assessment of resistance phenotype stability

To verify the phenotypic stability of the acquired resistance, the linezolid-induced resistant derivative of *S. aureus*_97 (designated LZR) was subcultured on drug-free Columbia blood agar plates. Serial passaging was performed for 50 consecutive passages, with each passage corresponding to 24 hours of incubation. The MIC was assessed every 10 passages using the broth microdilution method described above to determine whether the MIC values changed significantly.

### 2.5 Efflux pump inhibition assay

Fresh colonies of the LZR strain, which exhibited a linezolid MIC of 32 μg/mL, were selected from overnight cultures and used to prepare a bacterial suspension adjusted to a 0.5 McFarland standard. The efflux pump inhibitor carbonyl cyanide m−chlorophenylhydrazone (CCCP) powder (Shanghai Aladdin Biochemical Technology Co., Ltd., China; Lot No. A2614029) was dissolved in dimethyl sulfoxide (DMSO), and sterile nutrient broth was used as the diluent. Experimental groups were organized based on different CCCP concentrations, specifically 4 μg/mL and 2 μg/mL. A series of linezolid concentrations were prepared and dispensed into 96−well plates, followed by the addition of CCCP solution and bacterial suspension to each well. Three replicates were set for each group. The final concentrations in each well were as follows: CCCP at 4 μg/mL and 2 μg/mL, and linezolid at 256, 128, 64, 32, 16, 8, 4, 2, 1, 0.5, 0.25, and 0.125 μg/mL. A positive control group and a group containing only CCCP were also included. Bacterial growth was monitored by measuring OD600 every 2 hours during incubation at 37 °C for 16–20 hours using a SpectraMax Plus 384 microplate reader (Molecular Devices, United States). The minimum inhibitory concentration (MIC) was defined as the lowest drug concentration that completely inhibited bacterial growth. Changes in linezolid MIC were compared before and after CCCP addition.

### 2.6 RNA extraction, isolation, library construction, sequencing, and bioinformatic analysis of *S. aureus*_97 before and after linezolid induction

Total RNA was extracted from equal volumes of logarithmic-phase cultures of the parental *S. aureus*_97 (LSS) strain and the linezolid-induced resistant derivative (LZR) using Trizol reagent. The quantity and integrity of the extracted RNA were precisely assessed using an Agilent 2100 bioanalyzer (Agilent Technologies, USA). Only RNA samples meeting quality criteria were used for subsequent library construction, which was performed using a strand-specific method. Following library construction, initial quantification was conducted using a Qubit 2.0 Fluorometer, and libraries were diluted to 1.5 ng/µL. The insert size of each library was then determined using the Agilent 2100 bioanalyzer. Once the insert size was confirmed to meet expectations, the effective concentration of each library was accurately quantified by quantitative real-time PCR (qRT-PCR), with the criterion that the effective concentration exceeded 1.5 nM to ensure library quality. Following quality control, libraries that met the criteria were pooled based on their effective concentrations and the required sequencing output and subsequently subjected to high-throughput sequencing on an Illumina platform. Raw sequencing data were filtered, and sequencing error rates and GC content distribution were analyzed to obtain high-quality, clean reads for subsequent analyses. The number of reads mapped to each gene was quantified using featureCounts (v2.0.6). Differential expression analysis between the two groups (LZR vs. LSS) was performed using the DESeq2 R package (v1.42.0). Statistical analysis of differentially expressed genes in Kyoto Encyclopedia of Genes and Genomes (KEGG) pathways was conducted using the clusterProfiler software. Three independent biological replicates were performed for each experimental group.

### 2.7 Metabolite extraction, LC-MS/MS analysis, and data analysis of S. aureus_97 before and after linezolid induction

Bacterial cells from equal volumes of logarithmic-phase cultures of the parental *S. aureus*_97 (LSS) strain and the linezolid-induced resistant derivative were harvested by centrifugation in Eppendorf tubes. Metabolites were extracted by adding an aqueous 80% methanol solution. The mixture was snap-frozen in liquid nitrogen for 5 minutes, thawed on ice, vortexed for 30 seconds, and sonicated for 6 minutes. After centrifugation at 5000 rpm for 1 minute at 4 °C, the supernatant was collected, lyophilized to dryness, and subsequently reconstituted in 10% methanol solution prior to LC-MS/MS analysis. Pooled quality control (QC) samples were prepared by mixing equal volumes of all experimental samples and processed in parallel with the test samples to enable system conditioning and quality monitoring. Untargeted metabolomic analysis was performed using a Vanquish UHPLC system (Thermo Fisher, Germany) equipped with a Hypesil Gold column (100 × 2.1 mm, 1.9 μm) coupled to an Orbitrap Exploris™ 480 high-resolution mass spectrometer (Thermo Fisher, Germany). Peak extraction, quantification, and alignment across samples were conducted using XCMS software. Metabolites were identified based on parameters including a 10-ppm mass tolerance and adduct ion information, with reference to the NovoNovo high-quality MS/MS spectral database (NovoMetDB). Metabolite annotation was performed using the KEGG (https://www.genome.jp/kegg/pathway.html), HMDB (https://hmdb.ca/metabolites), and LIPID Maps (http://www.lipidmaps.org/) databases. Three independent biological replicates were performed for each experimental group.

### 2.8 Pathway enrichment analysis of differential genes and differential metabolites in *S. aureus*_97 before and after linezolid induction

Differential genes and differential metabolites were mapped to the KEGG pathway database to identify common pathways enriched in both datasets. The ggplot2 package in R was used to generate a combined metabolomic and transcriptomic KEGG enrichment bubble plot for the pathways co-enriched in both analyses. Common pathway terms identified in the metabolomic and transcriptomic enrichment results were selected, and the pathview package in R was employed to construct integrated KEGG pathway enrichment maps illustrating the shared pathways.

### 2.9 Statistical analysis

Statistical analysis and data visualization were performed using GraphPad Prism 9 software. Differential expression analysis between the two groups was conducted using the DESeq2 R package (v1.42.0), DEGs were filtered by the combined cutoff of adjusted p-value (padj) ≤ 0.05 and |log_2_FoldChange| ≥ 1, and KEGG pathway enrichment analysis of differentially expressed genes was performed using clusterProfiler (v4.8.1). Metabolite annotation was conducted using the KEGG, HMDB, and LIPID Maps databases. For multivariate statistical analysis, metabolomic data were first transformed using metaX software, followed by principal component analysis (PCA) and partial least squares discriminant analysis (PLS-DA). Variable importance in projection (VIP) values for each metabolite were subsequently calculated. For univariate analysis, the statistical significance of differences in metabolite abundance between groups was assessed using Student’s t-test, and fold change (FC) values were also determined. Volcano plots and bubble plots were generated using the ggplot2 package in R, and clustering heatmaps were created using the pheatmap package. Pearson correlation coefficients between differential metabolites were calculated using the cor() function in R, and statistical significance was assessed with the cor.mtest() function (*P* < 0.05 considered significant). Correlation plots were subsequently generated using the corrplot package. Metabolic pathway enrichment was determined based on the criterion x/n > y/N, with *P* < 0.05 indicating statistically significant enrichment. Integrated KEGG pathway enrichment maps for shared pathways were constructed using the pathview package.

## 3 Results

### 3.1 Successful construction and phenotypic confirmation of a linezolid-induced resistant MSSA strain

**Figure 1:**
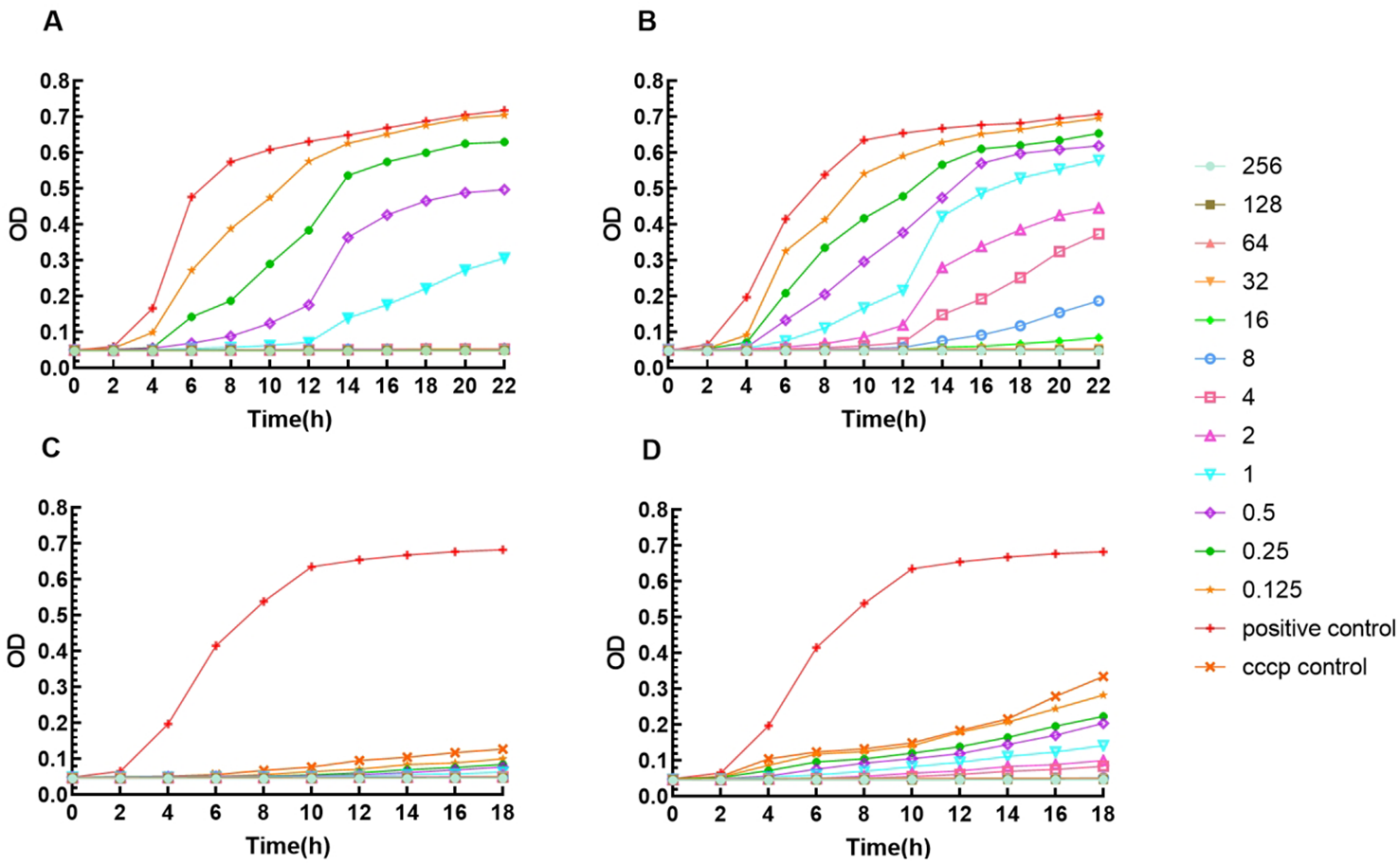
Growth curves of LSS and LZR under serially diluted linezolid. **(A)** LSS treated with gradient linezolid. **(B)** LZR treated with gradient linezolid. **(C)** LZR treated with 4 μg/mL CCCP plus gradient linezolid. **(D)** LZR treated with 2 μg/mL CCCP plus gradient linezolid. Positive control: drug-free group, CCCP control: group supplemented with CCCP only without linezolid.

Using a stepwise induction method with subinhibitory concentrations of linezolid, a stable linezolid-resistant derivative was successfully generated from a clinical linezolid-susceptible MSSA strain (*S. aureus*_97, initial MIC = 2 µg/mL). Antimicrobial susceptibility testing revealed that the MIC of linezolid for the induced *S. aureus*_97 (LZR) strain increased to 32 µg/mL, representing a 16-fold increase compared to the parental strain, thereby confirming the successful construction of the resistant strain (Caspers et al., 2017) (**Table 1**; **Figures 1A, B**). To assess the stability of the acquired resistance phenotype, the induced *S. aureus*_97 strain was serially passaged on Columbia blood agar plates without linezolid for 50 consecutive passages, with each passage corresponding to 24 hours of incubation. MIC values were re-determined every 10 passages and showed no significant changes over 50 passages, demonstrating that the linezolid resistance phenotype was stably inherited rather than a transient adaptive response.

**Table 1:** Dynamic changes in MIC of S.aureus_97 during *in vitro* linezolid resistance induction (0–36 passages, 0.5–64 μg/mL).

Passage number	Linezolid drug concentration (ug/mL)	S.aureus_97(LSS) MIC (ug/mL)	S.aureus_97(LZR) MIC (ug/mL)
0	0.5	2	2
4	1	2	2
8	2	2	2
12	4	2	2
16	8	2	4
20	16	2	8
24	32	2	16
28	64	2	32
32	64	2	32
36	64	2	32

### 3.2 The effect of the efflux pump inhibitor CCCP on the MIC of linezolid

Efflux pump inhibition assays revealed that the MIC of linezolid against LZR was markedly reduced after the addition of CCCP compared with that without CCCP treatment. A clear negative correlation was observed between CCCP concentration and linezolid susceptibility. When the CCCP concentration was decreased from 4 μg/mL to 2 μg/mL, the MIC of linezolid against LZR rose from 2 μg/mL to 8 μg/mL (**Figures 1C, D**).

### 3.3 Transcriptomic analysis of S. aureus_97 before and after linezolid induction

#### 3.3.1 Overview of RNA-seq data

**Figure 2:**
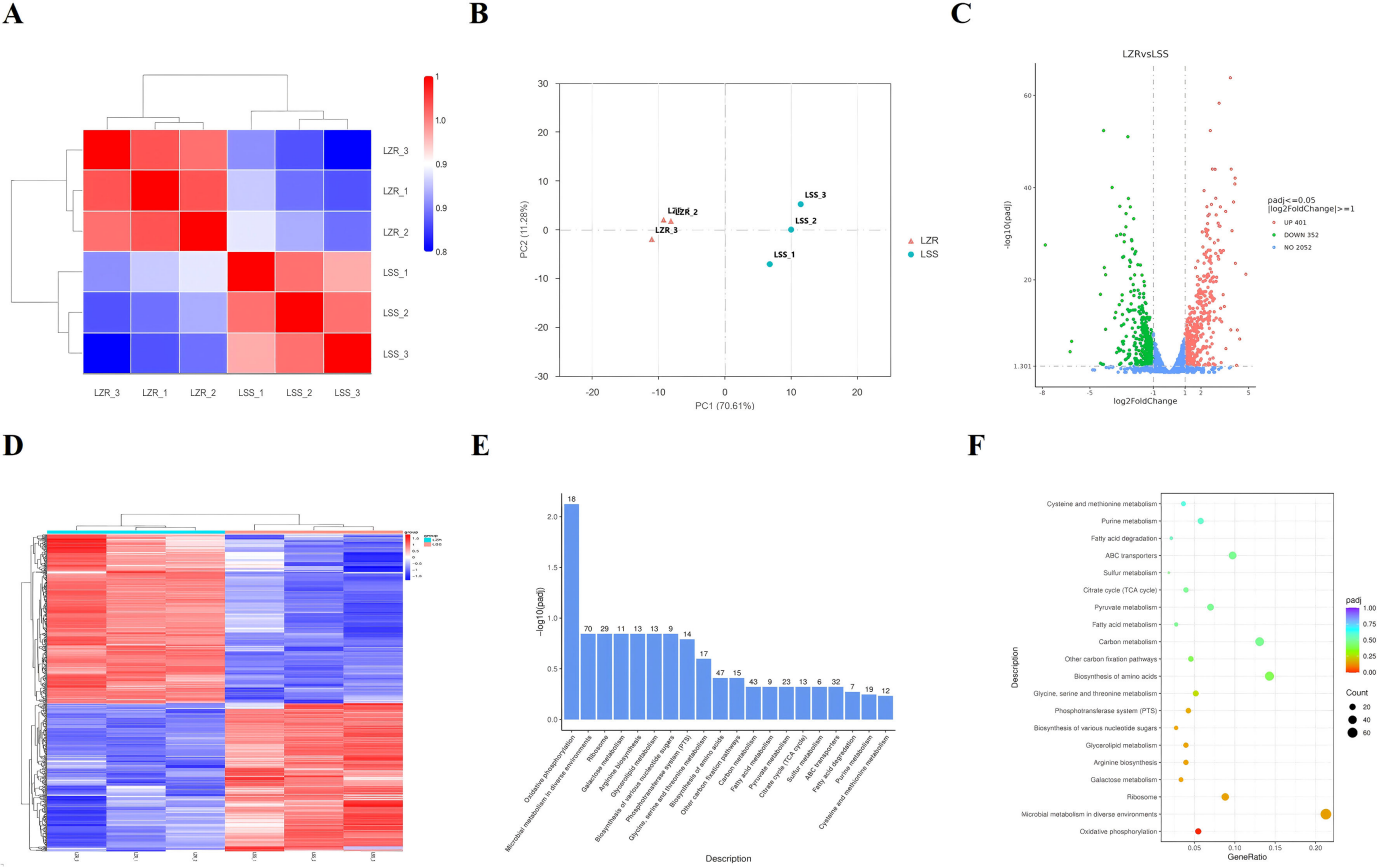
Transcriptomic profiling of (S) aureus 97 before and after linezolid induction (LZR group vs. LSS group). **(A)** Sample correlation heatmap based on Pearson correlation coefficients, showing the pairwise correlations among samples from the LZR group (n=3) and LSS group (n=3). A color gradient from blue to red indicates increasing correlation strength, with hierarchical clustering of samples illustrated on the axes. **(B)** Principal component analysis (PCA) score plot of the transcriptomic dataset. The first principal component (PC1) accounts for 70.61% of the total variance, while PC2 represents the secondary variance component. Each dot corresponds to an individual (S) aureus 97 sample (LZR group: red, LSS group: blue). **(C)** Volcano plot of differentially expressed genes (DEGs) between LZR and LSS groups. The x-axis denotes log_2_ (fold change) (LZR/LSS), and the y-axis represents -log_10_ (adjusted P-value, padj). Dots are colored by significance: red indicates significantly upregulated DEGs (padj ≤ 0.05 and log_2_FC ≥ 1); green indicates significantly downregulated DEGs (padj ≤ 0.05 and log_2_FC ≤ -1); blue indicates non-significant genes. **(D)** Hierarchical clustering heatmap of the 1321 identified DEGs. Rows represent genes and columns represent samples, with a color gradient from blue to red reflecting normalized gene expression levels (FPKM values). The top annotation bar indicates sample groups (LZR group: blue; LSS group: red). **(E)** Bar plot of KEGG pathway enrichment analysis for DEGs. The y-axis represents -log_10_(padj) as an indicator of enrichment significance, and the x-axis lists the enriched KEGG pathways. Numerical labels on the top of bars indicate the number of DEGs mapped to each pathway. **(F)** Bubble plot of KEGG pathway enrichment analysis for DEGs. The x-axis shows the GeneRatio (number of DEGs in a pathway/total number of genes in the pathway), and the y-axis lists KEGG pathways. The color intensity of bubbles represents padj values (red indicates higher significance), while the size of bubbles corresponds to the number of DEGs (Count) in each pathway.

The LZR and LSS groups yielded 7,641,142 and 7,731,367 clean reads, respectively, with approximately 97% of these reads successfully mapped to the *S. aureus* genome. Correlation analysis (**Figure 2A**) and PCA (**Figure 2B**) demonstrated good clustering of biological replicates within each group and a clear separation between the two groups, indicating high data quality and suitability for subsequent analyses.

#### 3.3.2 Key differentially expressed genes and their primary functions

Following linezolid induction, a total of 753 DEGs were identified in *S. aureus*_97. Compared to the LSS group, 401 genes were significantly upregulated, and 352 genes were significantly downregulated in the LZR group (**Figure 2C**; **Supplementary Table 1**). Hierarchical clustering analysis (**Figure 2D**) demonstrated that samples from the two groups clustered into distinct branches, indicating consistent and specific patterns of differential gene expression. The top 10 upregulated and downregulated genes were selected based on log2 fold change (LZR/LSS) (**Table 2**). Among these, the most significantly upregulated genes were those involved in ribosomal assembly, structural stability, and protein synthesis accuracy (*rpl* and *rps* families), followed by the gene encoding the core subunit of phenylalanyl-tRNA synthetase (*pheT*). The most significantly downregulated genes included the gene encoding the ribonuclease P subunit (*rnpA*), the gene encoding the catalytic subunit of aspartate carbamoyltransferase (*pyrB*), and the gene encoding a DNA repair regulator (*recX*).

**Table 2:** Top 10 up- and down-regulated differentially expressed genes (DEGs) in S. aureus 97 after exposure to linezolid (LZR group vs. LSS group).

Gene name	Regulation	Log_2_FC (LZR/LSS)	FDR	Annotation
rplR	Up	3.91	9.50E-45	50S ribosomal protein L18
rpsE	Up	3.88	6.96E-10	30S ribosomal protein S5
rpsN	Up	3.41	4.21E-27	30S ribosomal protein S14
rplN	Up	3.28	1.19E-26	50S ribosomal protein L14
rpmD	Up	3.21	4.21E-27	50S ribosomal protein L30
rpsH	Up	3.20	2.26E-32	30S ribosomal protein S8
rplE	Up	3.15	2.06E-32	50S ribosomal protein L5
rplF	Up	3.14	4.80E-59	50S ribosomal protein L6
rplL	Up	3.08	7.56E-26	50S ribosomal protein L7/L12
pheT	Up	3.08	2.41E-29	phenylalanyl-tRNA synthetase subunit beta
rnpA	Down	-1.25	5.34E-04	ribonuclease P
pyrB	Down	-1.13	1.63E-05	aspartate carbamoyltransferase catalytic subunit
recX	Down	-1.10	5.91E-05	recombination regulator RecX
nsaR	Down	-0.93	2.39E-05	nisin susceptibility-associated DNA-binding response regulator
nsaS	Down	-0.88	3.90E-04	nisin susceptibility-associated sensor histidine kinase
gpmA	Down	-0.84	4.92E-05	2%2C3-bisphosphoglycerate-dependent phosphoglycerate mutase
miaA	Down	-0.80	3.66E-03	tRNA delta(2)-isopentenylpyrophosphate transferase
recF	Down	-0.74	2.31E-04	recombination protein F
pyrC	Down	-0.70	3.69E-02	dihydroorotase
hemE	Down	-0.70	6.84E-04	uroporphyrinogen decarboxylase

DEGs were identified with padj ≤ 0.05 and |log2FC(LZR/LSS)| ≥ 1. Log_2_FC denotes the log_2_ fold change of gene expression in LZR relative to LSS (upregulation, positive values; downregulation, negative values).

#### 3.3.3 KEGG pathway analysis of DEGs

KEGG pathway enrichment analysis revealed that the oxidative phosphorylation pathway exhibited the highest level of enrichment significance, followed by microbial metabolism in diverse environments, the ribosome, galactose metabolism, arginine biosynthesis, glycerolipid metabolism, biosynthesis of various nucleotides, and the phosphotransferase system (PTS). Pathways such as glycine, serine, and threonine metabolism; amino acid biosynthesis; and carbon metabolism showed moderate enrichment significance (**Figure 2E, F**).

### 3.4 Metabolomic analysis of *S. aureus*_97 before and after linezolid induction

#### 3.4.1 Overview of metabolomic data

**Figure 3:**
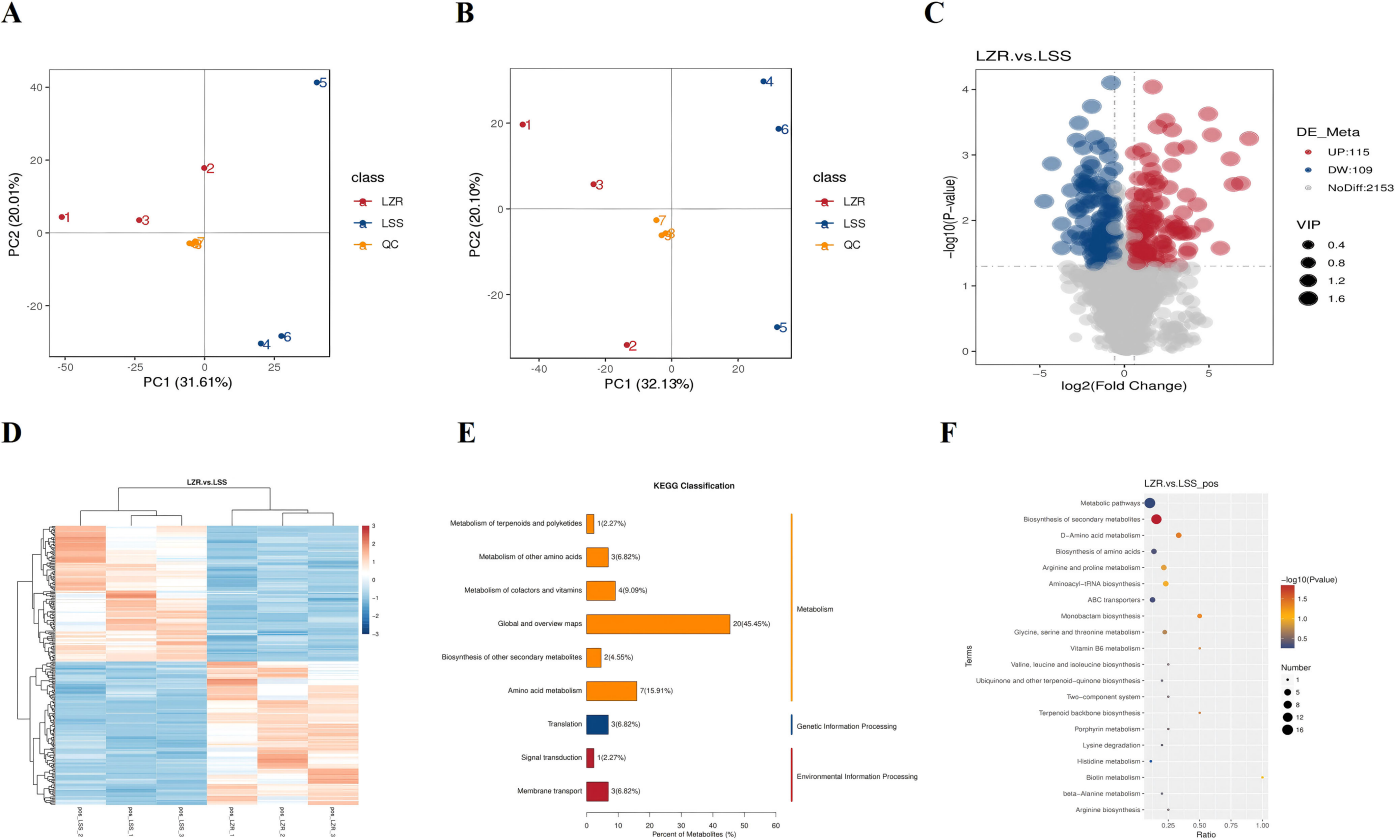
Metabolic profiling and functional annotation of (S) aureus 97 before and after linezolid induction (LZR group vs. LSS group). **(A)** PCA score plot in positive ion mode (PC1: 31.61%, PC2: 20.01%); red = LZR, blue = LSS, orange = QC. **(B)** PCA score plot in negative ion mode (PC1: 32.13%, PC2: 20.10%); color coding as in **(A)**. **(C)** Volcano plot of differential metabolites; red = upregulated (n=115), blue = downregulated (n=109), gray = non-differential (n=2153); dot size = VIP value, dashed line = P < 0.05. **(D)** Hierarchical clustering heatmap of differential metabolites; red/blue gradient indicates high/low metabolite abundance. **(E)** KEGG classification of differential metabolites; color-coded by KEGG primary categories (orange = Metabolism, blue = Genetic Information Processing, red = Environmental Information Processing). **(F)** KEGG pathway enrichment scatter plot of regulated metabolites (LZR.vs.LSS_pos); dot size = number of differential metabolites, color = -log10(P-value), x-axis = enrichment ratio.

PCA results obtained in positive and negative ion modes (**Figures 3A, B**) demonstrated a clear separation between the LZR and LSS groups. In both ion modes, the cumulative explained variance for principal components 1 and 2 reached 51.62% and 52.23%, respectively. The tight clustering of quality control (QC) samples confirmed the stability and reliability of the analytical run.

#### 3.4.2 Differentially abundant metabolites and their primary functions

Comparative analysis between the linezolid-induced LZR group and the pre-induction LSS group identified 224 DAMs, of which 115 were upregulated and 109 were downregulated (**Figure 3C**; **Supplementary Table 2**). To further elucidate the key metabolic alterations between the LZR and LSS groups, the top 10 upregulated and top 10 downregulated differentially abundant metabolites are summarized in **Table 3**. These differentially accumulated metabolites were primarily classified as lipids and lipid-like molecules, as well as organic acids and their derivatives. Hierarchical clustering analysis of the differentially abundant metabolites (**Figure 3D**) revealed that the LZR and LSS groups were separated into two distinct clusters, indicating group-specific expression patterns for these metabolites.

**Table 3:** Top 10 up- and down-regulated differentially accumulated metabolites (DAMs) in S. aureus 97 after exposure to linezolid (LZR group vs. LSS group).

Compound name	Regulation	Log2FC (LZR/LSS)	P value	Annotation
PA(18:3(6Z,9Z,12Z)/18:0)	Up	7.37	0.001	Lipids and lipid-like molecules
TACROLIMUS	Up	6.89	0.003	Phenylpropanoids and polyketides
Soyasaponin Bb	Up	6.44	0.003	Lipids and lipid-like molecules
Creatine	Up	6.27	0.001	Organic acids and derivatives
Karaconitine	Up	5.67	0.027	Lipids and lipid-like molecules
CREATININE	Up	5.17	0.000	Organic acids and derivatives
Procainamide 4-hydroxylamine	Up	4.95	0.000	Benzenoids
2,5-Dimethyl-3-isoamylpyrazine	Up	4.70	0.012	Organoheterocyclic compounds
Tryptamine	Up	4.30	0.006	Organoheterocyclic compounds
Cadiamine	Up	3.95	0.015	Alkaloids and derivatives
Nanchangmycin	Down	-4.74	0.005	Lipids and lipid-like molecules
L-Prolinamide, glycyl-N-(4-methyl-2-oxo-2H-1-benzopyran-7-yl)-	Down	-4.29	0.001	Organic acids and derivatives
N-arachidonoyl dihydroxypropylamine	Down	-3.70	0.011	Lipids and lipid-like molecules
Tiapride	Down	-3.70	0.026	Benzenoids
Methionyl-Proline	Down	-3.21	0.018	Organic acids and derivatives
Aegeline	Down	-3.16	0.003	Phenylpropanoids and polyketides
Rubescensin M	Down	-2.92	0.006	Lipids and lipid-like molecules
CENTCHROMAN	Down	-2.83	0.001	Phenylpropanoids and polyketides
Ile Gln Leu	Down	-2.81	0.007	Organic acids and derivatives
16,16-dimethyl-PGA2	Down	-2.81	0.015	Lipids and lipid-like molecules

Log_2_FC denotes the log_2_ fold change of metabolite abundance in LZR relative to LSS, with positive values indicating upregulation and negative values indicating downregulation. P value represents the statistical significance of differential expression, and Annotation refers to metabolite class annotations based on metabolomic databases.

#### 3.4.3 KEGG pathway enrichment analysis of DAMs

KEGG classification results indicated that 84.09% of the differentially abundant metabolites were assigned to the metabolism category, with the highest proportions observed in global and overview maps (45.45%) and amino acid metabolism (15.91%), followed by metabolism of cofactors and vitamins (9.09%) (**Figure 3E**). KEGG pathway analysis revealed that the biosynthesis of secondary metabolites was the most significantly enriched pathway. Besides, amino acid metabolism-related pathways, including D-amino acid metabolism and arginine and proline metabolism, exhibited moderate enrichment (**Figure 3F**).

### 3.5 Integrated analysis of transcriptomic and metabolomic data of *S. aureus*_97 before and after linezolid induction

#### 3.5.1 KEGG pathway analysis of differentially expressed genes and differentially abundant metabolites commonly enriched

**Figure 4:**
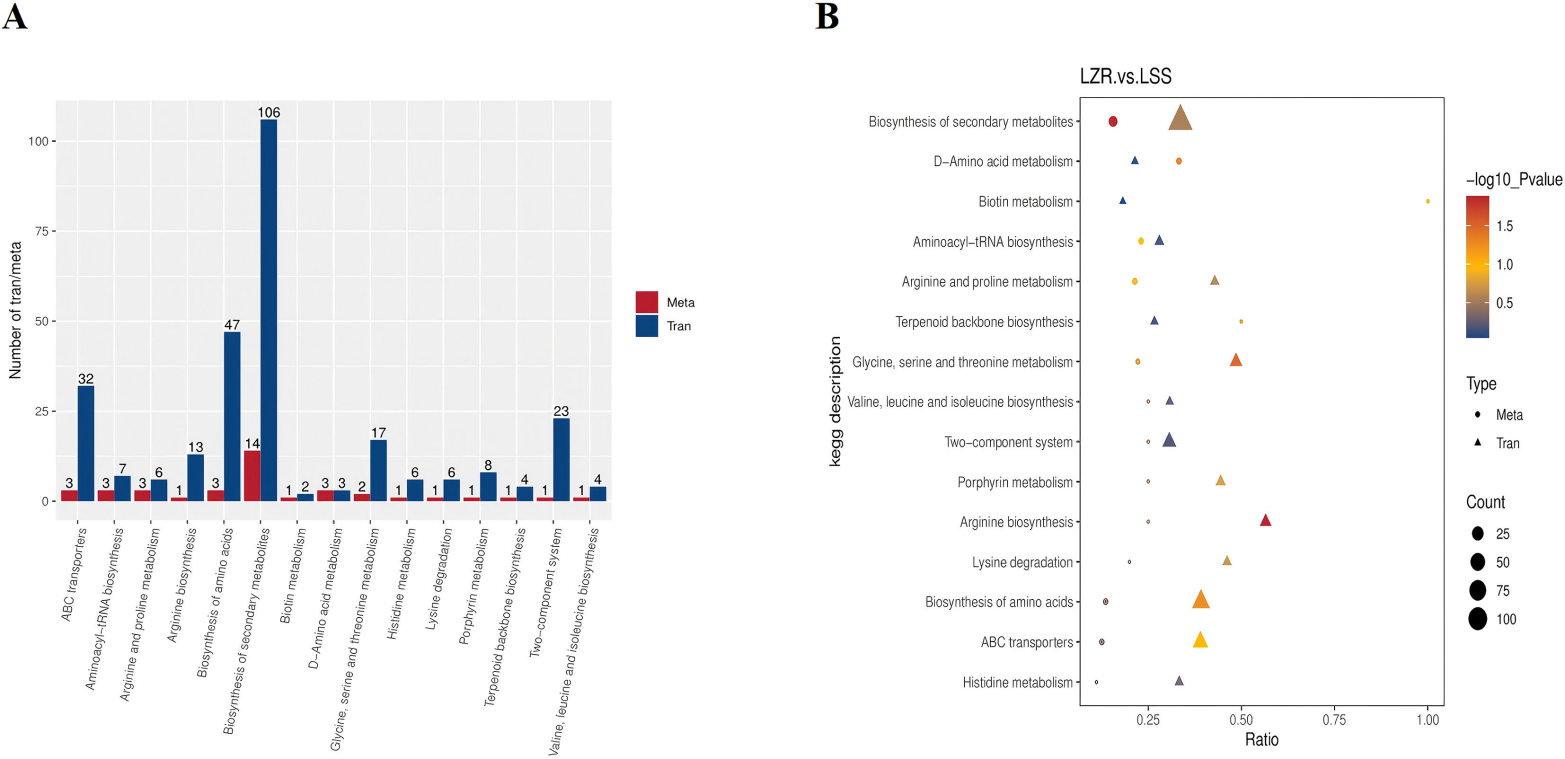
KEGG pathway enrichment analysis of transcriptomic and metabolomic differences of (S) aureus 97 before and after linezolid induction (LZR group vs. LSS group). **(A)** Bar chart showing the number of differentially expressed transcripts (blue bars) and metabolites (red bars) across KEGG pathways. **(B)** Bubble plot of KEGG pathway enrichment. The x-axis represents the ratio of differential genes/metabolites to total pathway genes/metabolites; the y-axis lists pathway names. Bubble color indicates enrichment significance (-log10(P-value)), size reflects the number of differential elements, and shape distinguishes transcriptomic (triangles) and metabolomic (circles) data.

KEGG pathway analysis revealed that DEGs and DAMs were co-enriched in 15 KEGG pathways (**Supplementary Table 3**). Among these, the secondary metabolite biosynthesis pathway exhibited the highest enrichment significance, with 106 transcripts enriched. D-Amino acid metabolism and biotin metabolism pathways also showed significant enrichment. Pathways such as arginine and proline metabolism, glycine-serine-threonine metabolism, valine-leucine-isoleucine biosynthesis, and arginine biosynthesis demonstrated moderate enrichment. In most pathways, the enrichment significance and corresponding bubble sizes in the bubble plot were more prominent for transcripts than for metabolites. Notably, the two-component system pathway contained a substantial number of differentially expressed transcripts but very few differentially abundant metabolites (**Figures 4A, B**).

#### 3.5.2 Differential expression of key enzymes in the secondary metabolite biosynthesis pathway

**Figure 5:**
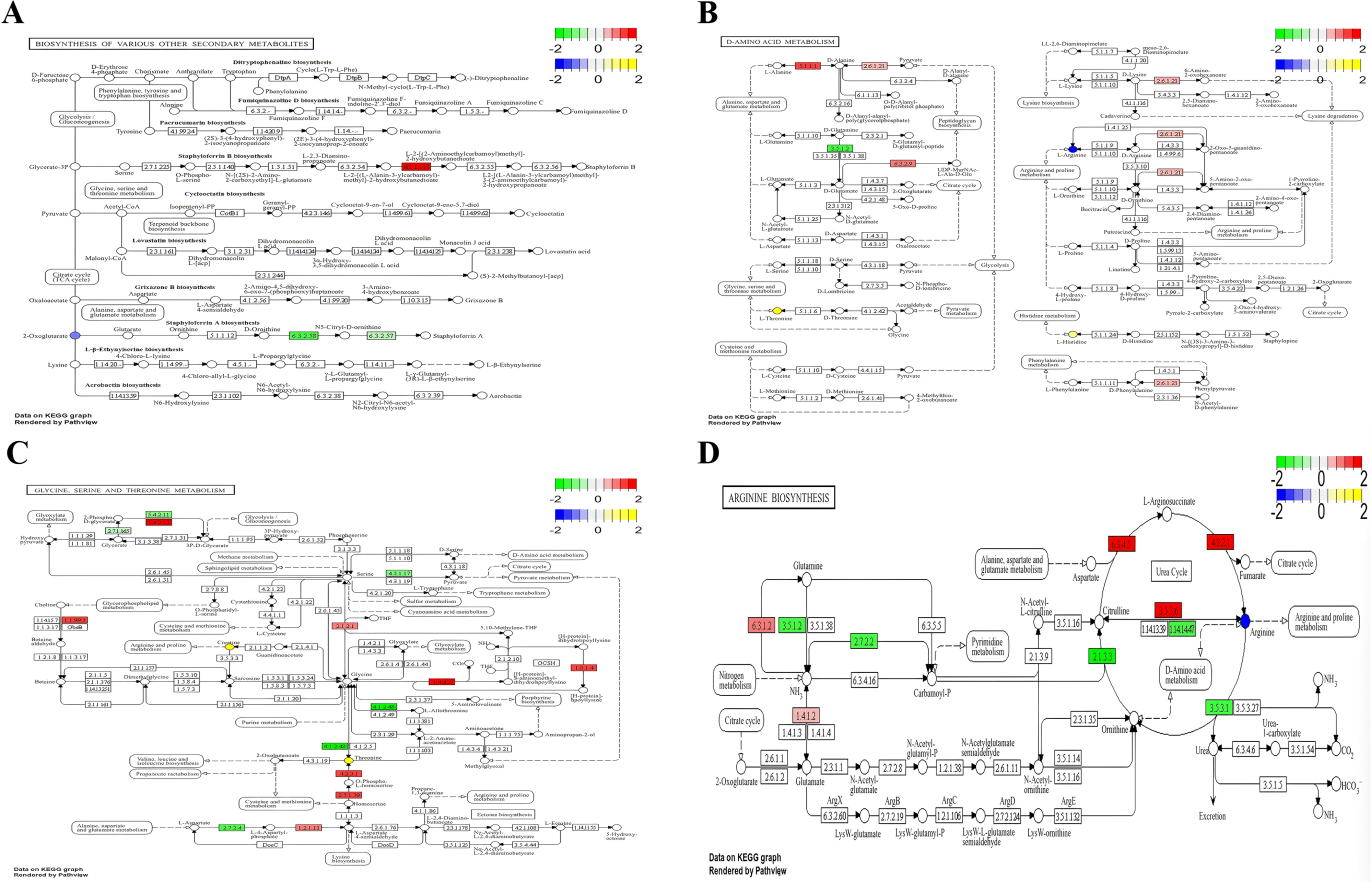
Pathview visualization of key metabolic pathways of (S) aureus 97 after linezolid induction. **(A)** Biosynthetic pathway of secondary metabolites. **(B)** D-amino acid metabolic pathway. **(C)** Glycine-serine-threonine metabolic pathway. **(D)** Arginine biosynthetic pathway. In the KEGG pathway maps, circles represent metabolites, with blue circles indicating downregulated differential metabolites and yellow circles indicating upregulated differential metabolites; boxes represent genes or proteins, where green boxes denote genes with significantly downregulated expression and red boxes denote genes with significantly upregulated expression.

Pathway visualization using the KEGG database and the Pathview tool revealed differential expression patterns within the biosynthesis pathway of various secondary metabolites in the LZR group compared to the LSS group. In the staphylococcal siderophore B biosynthetic branch, the node representing L-2,4-diaminobutyrate decarboxylase (EC 4.1.1.117) exhibited a deep red color, indicating marked upregulation in the LZR group and suggesting significant activation of this branch. In contrast, within the staphylococcal siderophore A biosynthetic branch, the nodes for N^5^-succinyl-L-ornithine: citrate ligase (EC 6.3.2.58) and N²-succinyl-L-ornithine: citrate ligase (EC 6.3.2.57) appeared green, demonstrating significant downregulation in the LZR group. Furthermore, the metabolite node representing the key precursor, 2-oxoglutarate, showed a pronounced downregulation in the LZR group (**Figure 5A**).

#### 3.5.3 Differential expression of key enzymes in the D-amino acid metabolism pathway

Pathway visualization of the KEGG D-amino acid metabolism pathway using the Pathview tool revealed distinct differential regulatory patterns in enzyme expression between the LZR and LSS groups. Specifically, the expression nodes for alanine racemase (EC 5.1.1.1) and UDP-N-acetylmuramoyl-L-alanyl-D-glutamate synthetase (EC 6.3.2.9) appeared deep red, indicating marked upregulation in the LZR group. The node for D-amino acid transaminase (EC 2.6.1.21) appeared light red and was significantly upregulated. In contrast, the expression node for asparaginase (EC 3.5.1.2) was deep green, reflecting pronounced downregulation in the LZR group. Analysis of metabolite nodes revealed that L-threonine and L-histidine were significantly increased in the LZR group, while L-arginine was markedly decreased (**Figure 5B**).

#### 3.5.4 Differential expression characteristics of key enzymes in the glycine-serine-threonine metabolism pathway

Pathway visualization of the KEGG glycine-serine-threonine metabolism pathway using the Pathview tool revealed distinct differential regulatory patterns in enzyme expression between the LZR and LSS groups. Specifically, the expression nodes for cofactor-independent phosphoglycerate mutase (EC 5.4.2.12), glycine dehydrogenase (EC 1.4.4.2), dihydrolipoamide dehydrogenase (EC 1.8.1.4), choline dehydrogenase (EC 1.1.99.1), threonine synthase (EC 4.2.3.1), and homoserine kinase (EC 2.7.1.39) appeared deep red, indicating marked upregulation in the LZR group. The nodes for glycine hydroxymethyltransferase (EC 2.1.2.1) and aspartate-semialdehyde dehydrogenase (EC 1.2.1.11) appeared light red, also demonstrating significant upregulation. In contrast, the expression nodes for 2,3-bisphosphoglycerate-dependent phosphoglycerate mutase (EC 5.4.2.11), L-serine dehydratase (EC 4.3.1.17), L-threonine aldolase (EC 4.1.2.48), and aspartate kinase (EC 2.7.2.4) were deep green, reflecting pronounced downregulation in the LZR group. The node for phosphoglycerate kinase (EC 2.7.1.165) appeared light green, indicating significant downregulation. Analysis of metabolite nodes revealed that creatine and threonine levels were markedly upregulated in the LZR group (**Figure 5C**).

#### 3.5.5 Differential expression characteristics of key enzymes in the arginine biosynthesis pathway

Pathway visualization of the KEGG arginine biosynthesis pathway using the Pathview tool revealed a bidirectional and distinct regulatory pattern, characterized by “activation of downstream synthesis coupled with inhibition of catabolic bypasses”, in the LZR group compared to the LSS group. Within the arginine synthesis pathway, the expression nodes for argininosuccinate synthase (EC 6.3.4.5), argininosuccinate lyase (EC 4.3.2.1), and arginase (EC 3.5.3.6) appeared deep red, indicating marked upregulation in the LZR group. The nodes for glutamine synthetase (EC 6.3.1.2) and glutamate dehydrogenase (EC 1.4.1.2) appeared light red, also demonstrating significant upregulation. In contrast, within arginine catabolism and bypass metabolic pathways, the expression nodes for glutaminase (EC 3.5.1.2), aspartate kinase (EC 2.7.2.2), carbamoyl-phosphate synthase I (EC 2.1.3.3), arginine N-monooxygenase (EC 1.14.14.47), and ornithine deaminase (EC 3.5.3.1) were deep green, reflecting pronounced downregulation in the LZR group. Analysis of metabolite nodes revealed that, despite marked upregulation of downstream synthetic enzymes, arginine levels remained significantly decreased in the LZR group (**Figure 5D**).

## 4 Discussion

Linezolid serves as a “last line of defense” in the clinical treatment of severe *S. aureus* infections; however, the emergence and spread of linezolid resistance have emerged as a critical challenge in current clinical anti-infective therapy. In this study, a linezolid-resistant MSSA strain was successfully constructed, and transcriptomic differences before and after linezolid induction were systematically compared using RNA-seq technology. The results showed that among the top 10 most significantly upregulated DEGs, ribosomal protein genes (*rpl* and *rps* families) were predominant. Independent biological RNA replicates were further applied for qRT-PCR validation of the key DEGs (rplR and rpsE). The high concordance between qRT-PCR results and transcriptomic data (**Supplementary Table 4**; **Supplementary Figure 1**) strongly validated the reliability and reproducibility of our RNA-seq dataset.

Linezolid exerts its antibacterial effect by binding to the peptidyl transferase center of the bacterial 50S ribosomal subunit, which blocks microbial protein synthesis. Bacteria develop resistance to these ribosome-targeting antibiotics primarily through target site alterations, including 23S rRNA mutation or methylation, as well as active drug efflux (Liu et al., 2024b). However, Sanger sequencing confirmed no mutations in the V domain of 23S rRNA and no acquired resistance genes (*cfr, optrA*) in the tested strains. In this context, the coordinated upregulation of ribosomal protein genes indicates a potential alternative adaptive resistance mechanism in bacteria. By elevating overall ribosome abundance and stabilizing ribosomal conformation, bacteria counteract linezolid-mediated ribosomal functional inhibition, sustain normal protein synthesis under antibiotic stress, and ultimately develop drug-resistant phenotypes (An et al., 2020).

Notably, linezolid-resistant strains displayed significant downregulation of *rnpA*, *pyrB*, and *recX*, revealing the adoption of a coordinated and multidimensional adaptive strategy against antibiotic stress. *rnpA* encodes the protein subunit of ribonuclease P, which catalyzes the 5′-terminal maturation and cleavage of pre-tRNA. The downregulation of this gene hinders intracellular RNA processing and degradation, resulting in RNA accumulation. It may compensatorily activate the protein synthesis system through competitive binding, thereby partially reversing the translational inhibition induced by linezolid (Chung et al., 2023; Suigo et al., 2021). *recX* functions as an essential negative modulator of RecA recombinase. Its diminished expression derepresses the SOS DNA repair cascade, augments bacterial tolerance to genotoxic damage, sustains genomic plasticity under prolonged antibiotic stress, and consequently drives the stable fixation of adaptive resistance mutations (Jung et al., 2025). *pyrB* encodes aspartate carbamoyltransferase, the rate-limiting enzyme of *de novo* pyrimidine biosynthesis. Downregulated *pyrB* expression signifies a metabolic reprogramming toward an energy-sparing survival state in resistant strains: the bacteria decelerate cellular proliferation and reallocate scarce carbon, energy and amino acid pools from growth-associated metabolism to stress-defense pathways, a metabolic signature closely analogous to the bacterial stringent response triggered by environmental adversity (Luo et al., 2025b).

Functionally, efflux inhibition assays verified that PMF-dependent efflux constitutes a critical contributor to linezolid resistance in LZR strains. The PMF inhibitor CCCP reverses the linezolid-resistant phenotype in a concentration-dependent fashion; specifically, lower CCCP concentrations lead to weaker efflux inhibition and enhanced bacterial resistance. Combined with stable mutations in the efflux gene *norA* identified by whole-genome sequencing (**Supplementary Table 5**), we speculate that mutation-mediated upregulation of efflux activity drastically reduces intracellular linezolid accumulation, which acts as a crucial mechanism sustaining the resistant phenotype (Sinha et al., 2024).

Integrated multi-omics analyses further confirm that linezolid resistance in this MSSA strain does not arise from single-gene variations alone; instead, it originates from systematic remodeling at the transcriptional and metabolic levels across the whole cell. Multiple core pathways in resistant strains, including oxidative phosphorylation, ribosome biogenesis, glycerolipid metabolism, amino acid metabolism and secondary metabolite biosynthesis, undergo concurrent remodeling. Multiple core pathways in resistant strains, including oxidative phosphorylation, ribosome biogenesis, glycerolipid metabolism, amino acid metabolism and secondary metabolite biosynthesis, undergo concurrent remodeling. Elevated oxidative phosphorylation furnishes sufficient ATP to fuel energy-consuming resistance-associated processes, including efflux pump operation, ribosomal remodeling and stress responses (Giddings et al., 2021; Liu et al., 2023; Back et al., 2026). Lipids and lipid-like molecules, as core structural components of the cell membrane, exhibit metabolic alterations that may impact the efficiency of intracellular linezolid uptake by modulating membrane fluidity, permeability, and the spatial conformation of lipid rafts (Balakrishnan and Kenworthy, 2024; Endapally et al., 2019). Reprogrammed amino acid metabolism and secondary metabolic pathways strengthen the strain’s tolerance to oxidative stress and sustain intracellular homeostasis under drug stress. Specifically, D-amino acids serve as essential precursors for peptidoglycan synthesis in the cell wall; disturbed metabolism of these compounds may modify cell wall architecture and permeability, restricting cellular entry of linezolid and attenuating its antibacterial efficacy (Miyamoto et al., 2020). The transcriptional upregulation of the arginine biosynthesis pathway may enhance the strain’s antibiotic tolerance by modulating stress-response protein expression (Reslane et al., 2024). Glycine-serine-threonine metabolism may contribute to global metabolic reprogramming under resistant conditions by modulating the efficiency of intracellular ATP generation (Ye et al., 2018). The activation of the phosphotransferase system (PTS) pathway may provide the material basis for metabolic remodeling in the resistant strain by optimizing carbon source utilization efficiency (Sánchez-Cañizares et al., 2020). Biotin acts as a vital cofactor governing multiple metabolic cascades, and its metabolic reprogramming facilitates fundamental survival and proliferation of bacteria under drug-triggered stress (Satiaputra et al., 2018; Shi et al., 2023). Branched-chain amino acids may mitigate drug-induced cellular injury by modulating intracellular osmotic homeostasis and enhancing oxidative stress tolerance, an adaptive mechanism analogous to survival strategies employed by tumor cells under stressful conditions (Cai et al., 2025).

Furthermore, in most co-enriched pathways, the enrichment significance of DEGs was markedly higher than that of DAMs, further supporting a hierarchical regulatory architecture in linezolid resistance, with transcriptional regulation as the primary driver and metabolic remodeling as a downstream effector. For instance, the two-component system pathway exhibited significant enrichment of DEGs, with no corresponding changes in DAMs, suggesting that this pathway does not directly participate in metabolite conversion but rather orchestrates the expression of key metabolic genes through transcriptional cascades, thereby indirectly mediating the development of the resistant phenotype. Combined with stable mutations in the two-component regulatory system *nsaS/nsaR* identified by whole-genome sequencing, we infer that structural alteration and altered signal transmission dynamics of this system constitute one central mechanism sustaining the resistant phenotype (Bhate et al., 2018; Truong et al., 2025).

As key functional molecules that enable bacteria to respond to environmental stress and maintain survival, secondary metabolites are dynamically regulated in their biosynthetic pathways, which constitutes an important mechanism in the development and evolution of bacterial resistance. Pathview visualization results revealed that in the LZR group, a key enzyme in the staphylococcal siderophore B biosynthetic pathway (L-2,4-diaminobutyrate decarboxylase) was markedly upregulated, suggesting specific activation of this pathway under linezolid stress. In contrast, two key rate-limiting enzymes in the staphylococcal siderophore A biosynthetic pathway (N^5^-succinyl-L-ornithine:citrate ligase and N²-succinyl-L-ornithine:citrate ligase) were significantly downregulated, indicating marked inhibition of siderophore A synthesis. This differential expression pattern suggests that *S. aureus* employs a “selective regulation” strategy for distinct siderophore types in response to linezolid resistance, rather than uniformly modulating the entire siderophore system. Previous studies have confirmed that siderophore B is better adapted to the iron-limited microenvironment under linezolid stress, and its upregulated synthesis rapidly meets the iron uptake requirements of the resistant strain. In contrast, the biosynthesis of siderophore A requires greater resources and energy consumption; under resource-limited conditions, inhibition of its synthesis helps reduce the bacterium’s overall metabolic burden. Therefore, the preferential synthesis of siderophore B coupled with the suppression of siderophore A represents an important adaptive survival strategy developed by the resistant strain under antibiotic pressure (Tang et al., 2019). Furthermore, as a key intermediate in the TCA cycle, the core precursor metabolite 2-oxoglutarate plays a crucial role in energy metabolism and various biosynthetic processes (Yuan et al., 2022; Legendre et al., 2022). The significantly decreased abundance of this metabolite in the LZR group reduced the overall efficiency of TCA cycle flux, further confirming that the resistant strain employs metabolic reprogramming to optimize energy allocation. By prioritizing the allocation of limited metabolic resources to resistance-associated pathways, the bacterium adapts to the drug-induced stressful environment.

Further KEGG enrichment analysis revealed that, in addition to secondary metabolism, D-amino acid metabolism, glycine-serine-threonine metabolism, and arginine biosynthesis were significantly remodeled through a bidirectional regulation pattern—activated anabolism coupled with suppressed catabolism—to optimize resource allocation under linezolid stress. Within the D-amino acid metabolism, upregulation of alanine racemase and UDP-N-acetylmuramoyl-L-alanyl-D-glutamate synthetase promoted D-alanine accumulation, enhancing cell wall stability and repair (Naorem et al., 2022; Prabhu et al., 2025; Dong et al., 2018). Elevated D-amino acid transaminase maintained amino acid homeostasis, while downregulated asparaginase reduced unnecessary degradation, ensuring sufficient substrate supply for cell wall biosynthesis (Bakunova et al., 2023; Sharma et al., 2025). Glycine-serine-threonine metabolism was reprogrammed to prioritize resistance-related anabolism. Altered phosphoglycerate mutase and downregulated phosphoglycerate kinase redirected energy flux toward stress adaptation (Zhang et al., 2024a). Upregulation of one-carbon unit and threonine synthesis provided precursors for nucleic acid synthesis and methylation, consistent with threonine accumulation (Xia et al., 2019; Pan et al., 2021). Increased choline dehydrogenase stabilized membrane integrity and reduced antibiotic permeability (Ma et al., 2024), whereas suppressed serine/threonine catabolism minimized precursor loss (Wang et al., 2021). Arginine metabolism followed a conserved synthesis-activation/catabolism-inhibition pattern. Upregulated biosynthetic enzymes and elevated arginase promoted arginine-to-ornithine conversion and polyamine production (Zhao et al., 2025; Niu et al., 2022). Coordinated downregulation of carbamoyl-phosphate synthase I, arginine N-monooxygenase, and ornithine deaminase reduced futile substrate consumption. This rewiring maximized flux toward polyamine-dependent antioxidant defense and translational homeostasis, establishing a high-turnover, low-reserve adaptive phenotype that facilitates linezolid resistance in *Staphylococcus aureus* (Chen et al., 2024; Reslane et al., 2022; Schibalski et al., 2024).

## 5 Conclusions

In this study, a stable linezolid-resistant derivative of the clinical MSSA strain *S. aureus*_97 was successfully generated using a stepwise induction method with subinhibitory concentrations of linezolid. The MIC of linezolid for the LZR strain increased from 2 µg/mL to 32 µg/mL, representing a 16-fold increase compared to the parental susceptible strain. Serial passaging for 50 generations in antibiotic-free medium confirmed the stable inheritance of the resistance phenotype. This work provides a robust *in vitro* model to investigate the mechanisms of linezolid resistance and demonstrates that the induction protocol effectively simulates the evolutionary process of bacterial resistance in clinical settings, with strong clinical relevance.

Linezolid serves as a critical therapeutic agent for severe Gram-positive bacterial infections, and the spread of linezolid resistance has emerged as a significant challenge in clinical anti-infective therapy. In this study, integrated analysis of transcriptomic (RNA-seq) and untargeted metabolomic data systematically elucidated a multi-layered regulatory network underlying linezolid resistance in MSSA. At the transcriptional level, the resistant strain exhibited enhanced ribosomal function (upregulation of *rpl* and *rps* family genes) as a core feature, coupled with increased amino acid activation efficiency (upregulation of pheT), while simultaneously downregulating non-essential metabolic processes such as RNA processing and DNA repair, resulting in global gene expression remodeling adapted to drug pressure. At the metabolic level, the resistant strain established a resistance adaptation mechanism characterized by “core function enhancement, auxiliary process adaptation, and metabolic network coordination” through lipid metabolic reprogramming (modulation of membrane composition and permeability), synergistic optimization of amino acid metabolic pathways (bidirectional regulation of D-amino acid metabolism, arginine biosynthesis, and related pathways), and energy metabolism remodeling (enrichment of the oxidative phosphorylation pathway).

Integrated transcriptomic and metabolomic analyses further confirmed that the secondary metabolite biosynthetic pathway serves as a central hub within the resistance regulatory network. This pathway enables adaptation to iron-limited environments by selectively regulating staphylococcal siderophore synthesis. Meanwhile, the synergistic mode of “activation of synthesis coupled with inhibition of catabolism” observed in amino acid metabolism pathways not only provides the material basis for cell wall repair and ribosome synthesis but also alleviates drug-induced oxidative damage and translational inhibition via polyamine production, reflecting cross-omics synergistic regulatory characteristics. Furthermore, the resistant strain constructed in this study exhibited high-level resistance with an MIC of 32 µg/mL, suggesting that inappropriate clinical use of linezolid may accelerate the evolution of linezolid resistance in MSSA. This finding underscores the importance of prudent antimicrobial stewardship and routine resistance surveillance.

In summary, this study comprehensively elucidated the adaptive mechanisms underlying linezolid resistance in MSSA from phenotypic, transcriptomic, and metabolomic perspectives, thereby providing a theoretical basis and experimental foundation for clinical resistance surveillance and the development of novel anti-infective strategies.

## Data Availability

The datasets presented in this study can be found in online repositories. The names of the repository/repositories and accession number(s) can be found in the article/**Supplementary Material**.
