# Supplementary figures and images for: Construction of a linezolid-resistant strain of methicillin-susceptible Staphylococcus aureus and its multi-omics based mechanism study
Source: Front Cell Infect Microbiol. 2026 Jun 29;16:1839912. doi: 10.3389/fcimb.2026.1839912 (PMC13357757; doi:10.3389/fcimb.2026.1839912)

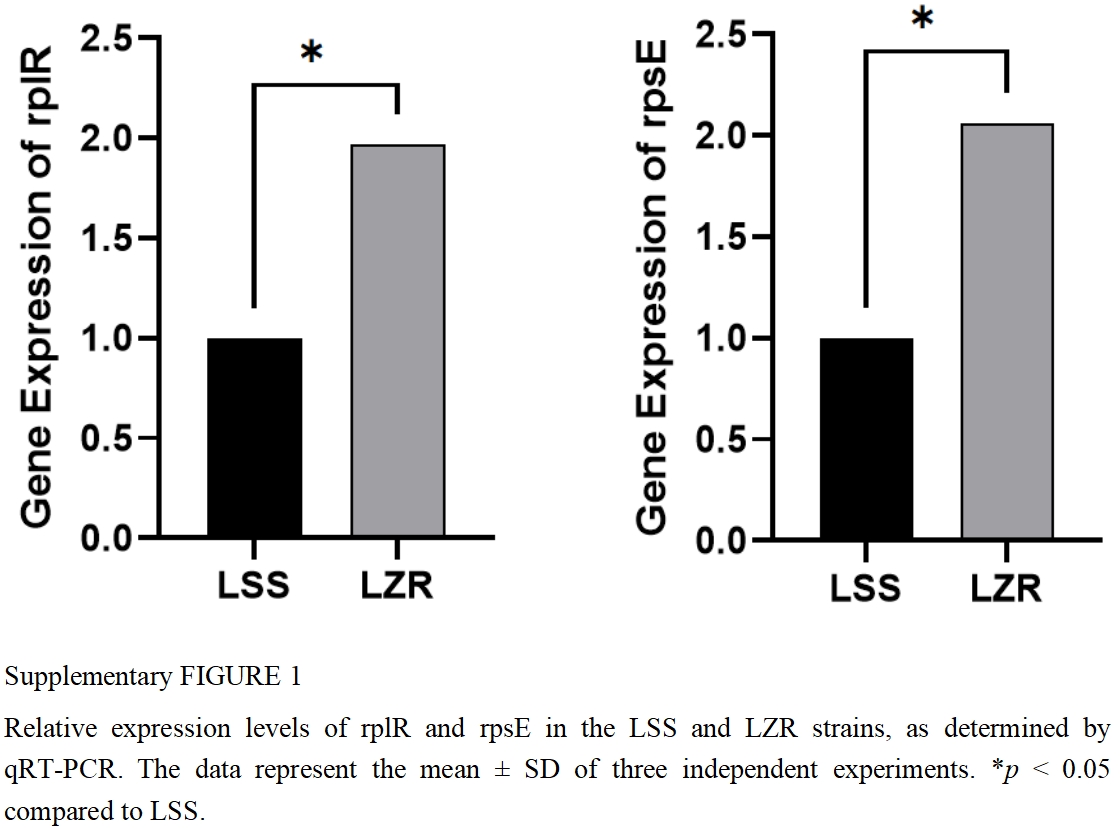

Supplement: Supplementary file 1 [file Image1.jpeg]

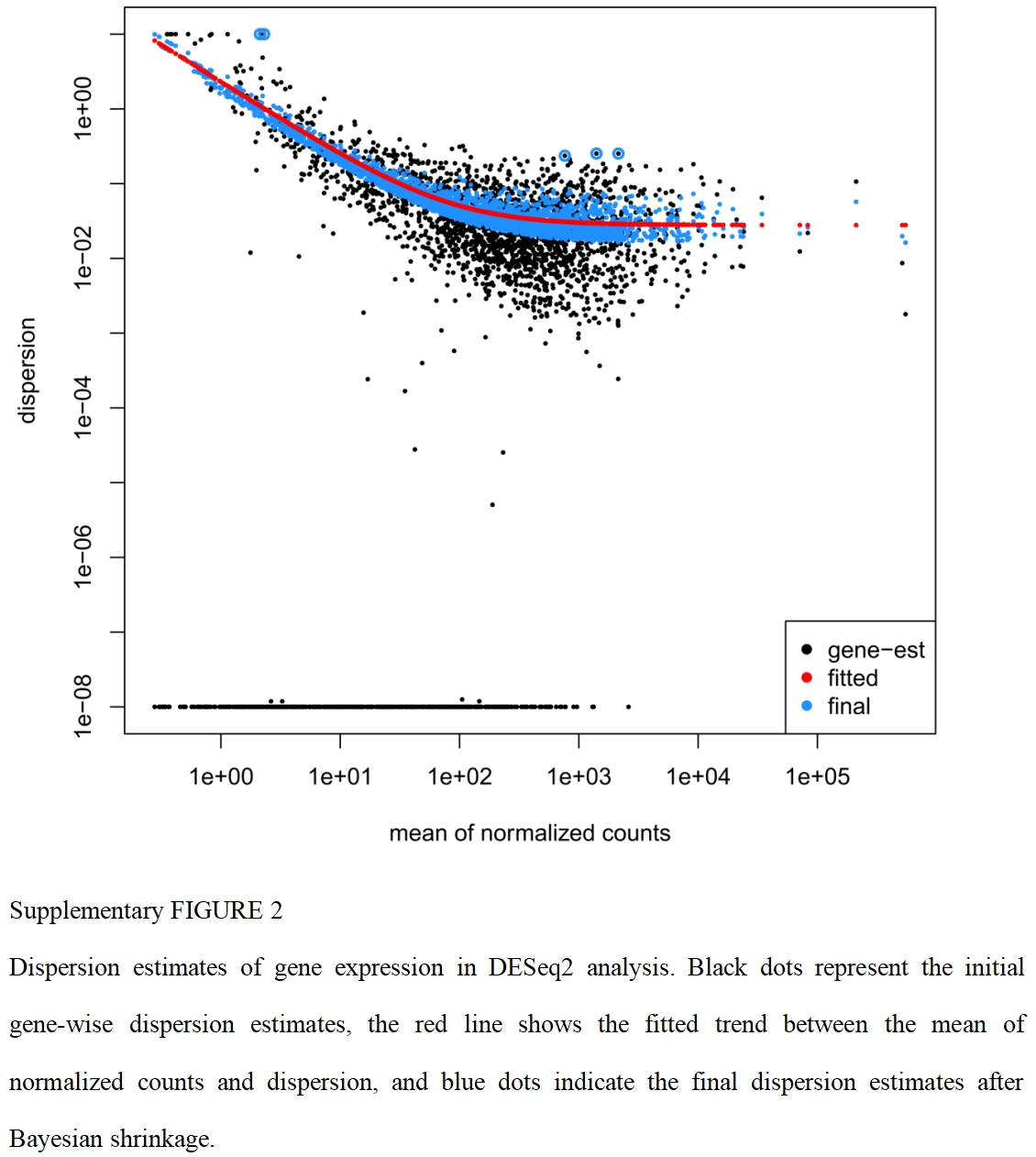

Supplement: Supplementary file 2 [file Image2.jpeg]
